# Dr. Ravin on the Papillæ of the Tongue

**Published:** 1826-07

**Authors:** 


14. In the Journal Universel des Sciences Medicales for November last, Dr. François Prosper Ravin, has offered some observations on the papillæ of the tongue, and on the symptomatology of this organ.

He differs from those anatomists who admit three orders of papillæ, and agrees with Heister in uniting the lenticulares and fungiformes under one head. He remarks, that with the exception of Winslow and Senac, most authors have erred in limiting these papillæ to the posterior part of the tongue, when in fact they are to be found in every part of its upper surface. They present, as Ruysh has stated, numerous small excretory apertures. They are placed symmetrically amongst the conical papillæ, but are fewest about the median line, and most conspicuous towards the sides. They are smaller, and situated with less regularity, but more thickly, towards the tip, where they are at least as numerous as the conical.

To the conical papillæ belongs all that part of the surface of the tongue, which is not occupied by the former kind. These papillæ are most numerous towards the raphe. For the most part they are conical and acute, but some along the sides of the tongue are flattened. A few terminate by a simple orifice for the escape of the fluid which they secrete, but the greater number end in short and slender filiform tubes, which Verheyen and Ravin have occasionally found double.

Dr. Ravin considers the first order of papillæ, viz. the lenticular and fungiform, as destined to secrete a fluid having the properties of saliva. To the conical he attributes the production of mucus. He conceives that the sympathies of these two orders of papillæ must be different, and he suggests that the knowledge of their peculiarities, would be an important assistance in diagnosis.

The conical papillæ often feel the influence of a slight gastro-intestinal, or bronchial irritation, and become concealed under a white fur, while the lenticular, whose secretion is uninterrupted, remain clean and conspicuous. These last, the author considers as more particularly affected by the state of the nerves. Hence, when the disorder is severe, when the nervous irritation and suffering are great, or when the membranes of the brain, and particularly the arachnoid about the origins of the nerves is affected, the lenticular papillæ either become red, or swollen, or their secretion is suspended. This influence first becomes visible towards the point of the tongue, where the nerves are most thickly distributed, and extends backwards in proportion to the intensity of the cause.

